# One-Year Follow-Up of Corneal Biomechanical Changes After Accelerated Transepithelial Corneal Cross-Linking in Pediatric Patients With Progressive Keratoconus

**DOI:** 10.3389/fmed.2021.663494

**Published:** 2021-07-07

**Authors:** Weijun Jian, Mi Tian, Xiaoyu Zhang, Ling Sun, Yang Shen, Meiyan Li, Xingtao Zhou

**Affiliations:** ^1^Department of Ophthalmology and Optometry, Eye and ENT Hospital, Fudan University, Shanghai, China; ^2^Key Laboratory of Myopia, Chinese Academy of Medical Sciences, Shanghai, China; ^3^Shanghai Research Center of Ophthalmology and Optometry, Shanghai, China

**Keywords:** pediatric, keratoconus, accelerated transepithelial cross-linking, Corvis ST, corneal biomechanics

## Abstract

**Aims:** This study aimed to investigate the corneal biomechanical changes and topographic outcomes of accelerated transepithelial corneal cross-linking (ATE-CXL) in pediatric progressive keratoconus.

**Methods:** In this prospective longitudinal study, 31 eyes of 28 pediatric patients with keratoconus (21 boys and 7 girls; mean age, 14.35 ± 2.68 years) undergoing ATE-CXL (epithelium-on procedure with 45 mW/cm^2^ for 320 s) were included. Corvis ST was used to measure dynamic corneal response parameters at baseline and at 12 month after ATE-CXL. Corneal keratometry and corneal thickness were measured using Pentacam pre-operatively and 1, 6, and 12 month post-operatively.

**Results:** No serious complications occurred during or after ATE-CXL. The maximum keratometry values were 60.10 ± 7.51 D pre-operatively and 61.42 ± 8.92, 61.17 ± 7.96, and 60.02 ± 7.58 D at 1, 6, and 12 month after ATE-CXL (*P* > 0.05), respectively. Corneal thickness remained stable during the 12-month follow-up (*P* > 0.05). At post-operative 12 month, first applanation time (*P* < 0.001), first applanation length (*P* = 0.004), second applanation velocity (*P* = 0.014), highest concavity time (*P* = 0.022), and radius of curvature at highest concavity (*P* = 0.031) increased significantly. The value of stiffness parameter at first applanation was significantly increased from 57.70 ± 27.57 pre-operatively to 63.36 ± 27.09 at 12 months after ATE-CXL (*P* = 0.018).

**Conclusions:** ATE-CXL is safe and effective in stabilizing the progression of pediatric keratoconus. Changes in corneal biomechanical response consistent with stiffening following ATE-CXL were observed in pediatric patients with keratoconus.

## Precis

ATE-CXL is safe and effective in stabilizing the progression and the corneal biomechanical properties of pediatric keratoconus.

## Introduction

Keratoconus is a bilateral progressive non-inflammatory ectatic corneal dystrophy characterized by thinning and steepening of the paracentral cornea (1), which usually appears during puberty, and early adulthood. Keratoconus seems to progress faster and to be more advanced at the time of diagnosis in pediatric patients compared to adults (2). Therefore, more attention should be paid to the treatment of pediatric keratoconus at early stages of the disease (3).

Corneal cross-linking (CXL) is considered an effective treatment for halting or reducing the progression of keratoconus with increasing corneal biomechanical stiffness. The majority of published studies (4–7) have demonstrated the safety and efficacy of CXL for pediatric keratoconus; however, most of these studies focused on conventional corneal cross-linking (C-CXL; epithelium-off, 3 mW/cm^2^ for 30 min) in the corneas of pediatric patients. Accelerated transepithelial corneal cross-linking (ATE-CXL; epithelium-on, 45 mW/cm^2^ for 320 s) (8–11) has several benefits over the conventional method such as epithelium-on procedure, less treatment time, less intraoperative and post-operative complications, and greater comfort for the patients. We were the first to report [in a previous study (10, 11)] the long-term safety and efficacy of ATE-CXL for progressive pediatric keratoconus.

Abnormal corneal biomechanical behavior is the most probable etiologic factor for keratoconus, thereby resulting in corneal morphologic changes (12). According to previous studies, the reduction of corneal biomechanical properties plays an important role in the progression of keratoconus. Corvis ST (13, 14) (OCULUS Optikgeräte GmbH; Wetzlar, Germany) is a non-contact tonometer that uses an ultra-high speed Scheimpflug camera to directly observe the response of the cornea to an air pressure pulse and provides a series of dynamic corneal response (DCR) parameters related to cornea deformation and corneal stiffness *in vivo*.

Several published studies have reported the use of Corvis ST in assessing the biomechanical properties of the cornea and in the diagnosis and treatment of keratoconus (15, 16). A previous study (17) has suggested early changes in biomechanics following CXL by Corvis ST in adult patients with keratoconus. However, studies that assess the biomechanical properties before and after ATE-CXL in pediatric patients with keratoconus are still lacking. Therefore, the aim of this study was to investigate the corneal biomechanical changes and topographic outcomes of ATE-CXL in pediatric patients with progressive keratoconus.

## Patients and Methods

### Patients

In this prospective study, we enrolled pediatric patients (aged 8–17 years) who were diagnosed with progressive keratoconus at the Eye and ENT Hospital of Fudan University in Shanghai, China. Evidence of progressive keratoconus included an increase in the maximum keratometry (Kmax) value or astigmatism >1 D in 1 year. Patients were excluded from the study if they had any of the following: stromal scarring in the cornea; worn rigid gas permeable lenses or soft contact lenses more than 4 or 2 weeks, respectively; a history of other ocular diseases that could affect corneal morphology; previous ocular surgeries; or allergy to riboflavin. We analyzed 31 eyes (24 boys and 7 girls, 14 right eyes and 17 left eyes) of 28 patients with a mean age of 14.35 ± 2.68 years. The baseline characteristics of the study population are shown in Table 1.

**Table 1:** Demographic data of the patients (Mean ± SD).

	**Mean ± SD**
Age (year)	14.35 ± 2.68
Sphere (D)	−6.04 ± 5.31
Cylinder (D)	−5.10 ± 2.15
SE (D)	−8.59 ± 5.89
CDVA (logMAR)	0.41 ± 0.32
K1 (D)	48.07 ± 4.94
K2 (D)	52.74 ± 5.07
Kmax (D)	60.10 ± 7.51
CCT (μm)	479.10 ± 45.33
AT (μm)	472.13 ± 45.83
TCT (μm)	461.55 ± 45.83

*D, diopters; SE, spherical equivalent; CDVA, corrected distance visual acuity; K1, steepest meridian keratometry; K2, flattest meridian keratometry; Kmax, maximum keratometry; CCT, central corneal thickness; AT, apex thickness; TCT, thinnest corneal thickness*.

This study was approved by the Ethics Committee of the Eye and ENT Hospital of Fudan University and was carried out following the tenets of the Declaration of Helsinki. One parent or legal guardian of each subject was provided with informed consent after a detailed explanation of the procedure prior to treatment.

### Measurements

The central corneal thickness (CCT), thinnest corneal thickness (TCT), apex thickness (AT), steepest meridian keratometry (K1), flattest meridian keratometry (K2), and Kmax values were obtained *via* Pentacam (OCULUS Optikgeräte GmbH; Wetzlar, Germany). All measurements with the Pentacam were operated by the same experienced technicians. The data were collected pre-operatively and at 1, 6, and 12 month after ATE-CXL.

DCR parameters were measured by the Corvis ST. The Corvis ST can capture series images of cornea by using the ultra-high-speed Scheimpflug camera while consistent air puff deforms the cornea. The definitions of the DCR parameters are listed in Table 2. Stiffness parameter at first applanation (SP-A1) was defined as the resultant pressure on the cornea divided by the corneal displacement in the first applanation position, which is considered to be a sensitive parameter for assessing corneal stiffness (18). A higher SP-A1 value indicates that the cornea has a stiffer response. All measurements with the Corvis ST were operated by the same experienced technicians. All patients were followed-up at post-operative 12 month.

**Table 2:** The meaning of Corvis ST parameters.

**Parameter**	**Explanation**
First applanation time (AT1)	Time to reach first applanation
First applanation length (AL1)	Length of the deformed part of the cornea at first applanation
First applanation velocity (AV1)	Corneal velocity at first applanation
Second applanation time (AT2)	Time to reach second applanation
Second applanation length (AL2)	Length of the deformed part of the cornea at second applanation
Second applanation velocity (AV2)	Corneal velocity at second applanation
Highest concavity time (HCT)	Time to reach highest concavity
Deformation amplitude (DA)	Sagittal length of deformation of the apex at highest concavity
Peak distance (PD)	The distance of the part of the cornea without deformation at highest concavity
Radius of curvature (Rad)	Radius of curvature at highest concavity
Deformation amplitude ratio (DAR)	The ratio between the deformation amplitude at the apex and the deformation amplitude at 2 mm
Integrated radius (Integr Rad)	The integrated area under the inverse radius curve during the concave phase
Stiffness parameter at first applanation (SP-A1)	Pressure at first applanation–Biomechanically corrected intraocular pressure/deflection amplitude at first applanation
Ambrósio's relational thickness horizontal (ARTh)	The division between corneal thickness at the thinnest point and the progression index that describes the thickness increase from the thinnest point to the periphery

### Cross-Linking Technique

All operations were performed by the same surgeon (X Zhou). ATE-CXL was initiated under topical anesthesia using oxybuprocaine hydrochloride eye drops. The cornea was treated by using ParaCel solution (0.25% riboflavin and benzalkonium chloride, Avedro) in the trephine (66 vision Tech, China) for 4 min, and then Vibex Xtra solution (0.25% riboflavin solution, Avedro) was dripped into the trephine for 6 min. After that, the cornea was rinsed with a balanced salt solution. Subsequently, the cornea was then exposed to 370 nm ultraviolet-A (UVA) light with the Avedro's KXL System (Avedro, Inc) for 5 min and 20 s at an irradiance level of 45 mW/cm^2^ in pulsed mode (1 s on, 1 s off). A balanced salt solution was applied to protect the cornea from dehydration during the irradiation. At the end of the procedure, the eye was patched with a bandage contact lens, and the contact lens was removed after 3 days until epithelial healing was complete. Post-operatively, 0.1% fluorometholone was prescribed seven times per day, and the frequency was gradually tapered down over 2 weeks. Additionally, topical antibiotic (levofloxacin) and artificial tears were prescribed for four times per day for a week and month, respectively.

### Data Analysis

Statistical analysis was performed in SPSS software (version 23; SPSS, Inc., Chicago, IL, USA) and SAS (version 9.4; SAS Institute, Cary, NC, USA). The results were analyzed using the Wilcoxon rank-sum test and repeated measures analyses of variance with Bonferroni-adjusted *post-hoc* comparisons. Inter-eye correlation was adjusted using a linear mixed model with random intercept to compare preoperative and post-operative parameters. Since certain DCR parameters were influenced by intraocular pressure (IOP) (19), it was included as a covariate in the model. Each follow-up result was compared with the preoperative values. *P* <0.05 was considered statistically significant.

## Results

All surgical procedures were completed successfully without any intraoperative or post-operative complications.

### Corneal Keratometry

**Figure 1:**
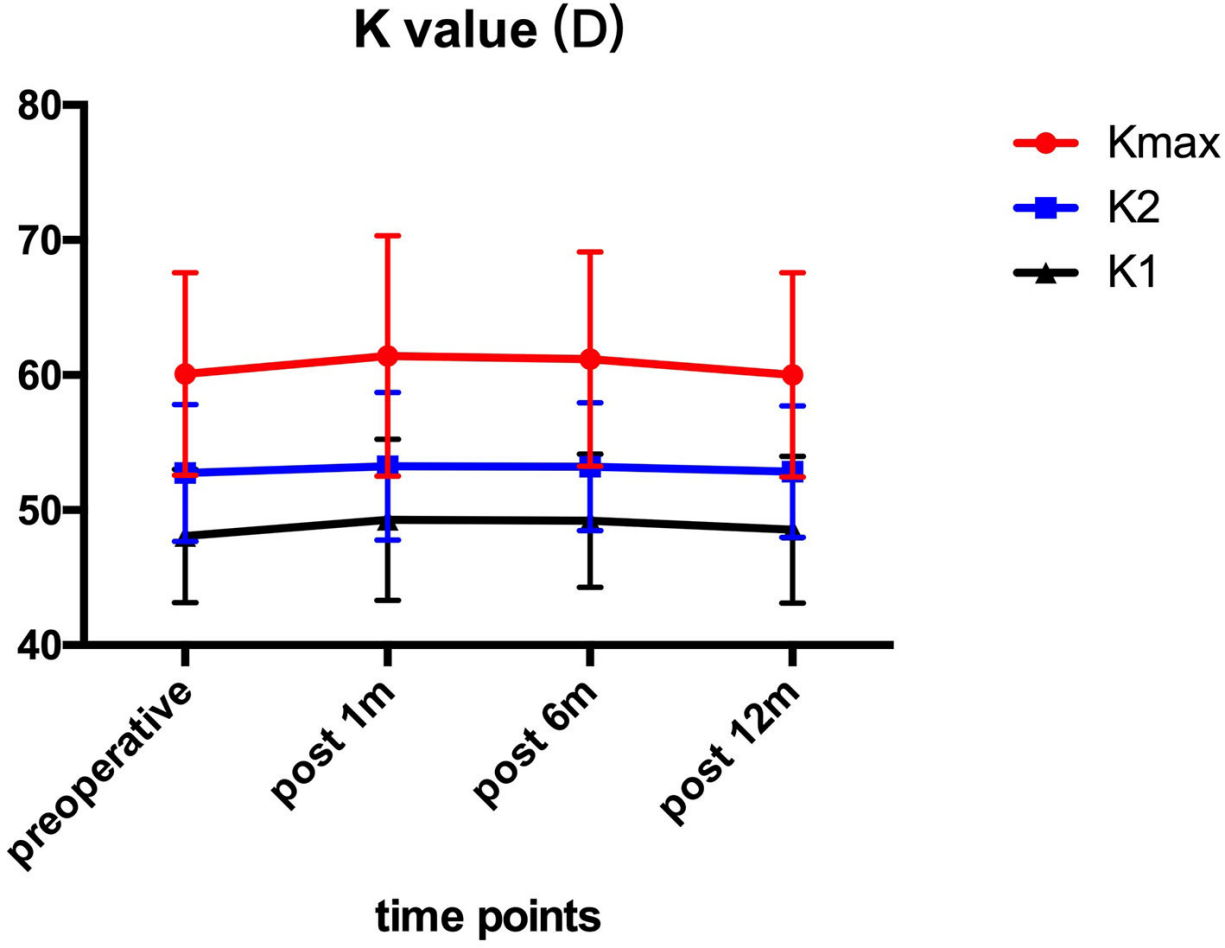
K1, K2, and Kmax before and after ATE-CXL during 1-year follow-up. There was no statistically significant (*P* > 0.05) alteration at each visit in comparison to preoperative values. (K1, steepest meridian keratometry; K2, flattest meridian keratometry; Kmax, maximum keratometry; ATE-CXL, accelerated transepithelial corneal cross-linking).

Pre- and post-operative corneal keratometry values are presented in Figure 1. The Kmax values were 60.10 ± 7.51 D preoperatively, and 61.42 ± 8.92, 61.17 ± 7.96, 60.02 ± 7.58 D at 1, 6, and 12 month after the operation. All patients showed no significant (*P* > 0.05) changes in K1, K2, and Kmax values in comparison to preoperative values at each follow-up time point.

### Corneal Thickness

**Figure 2:**
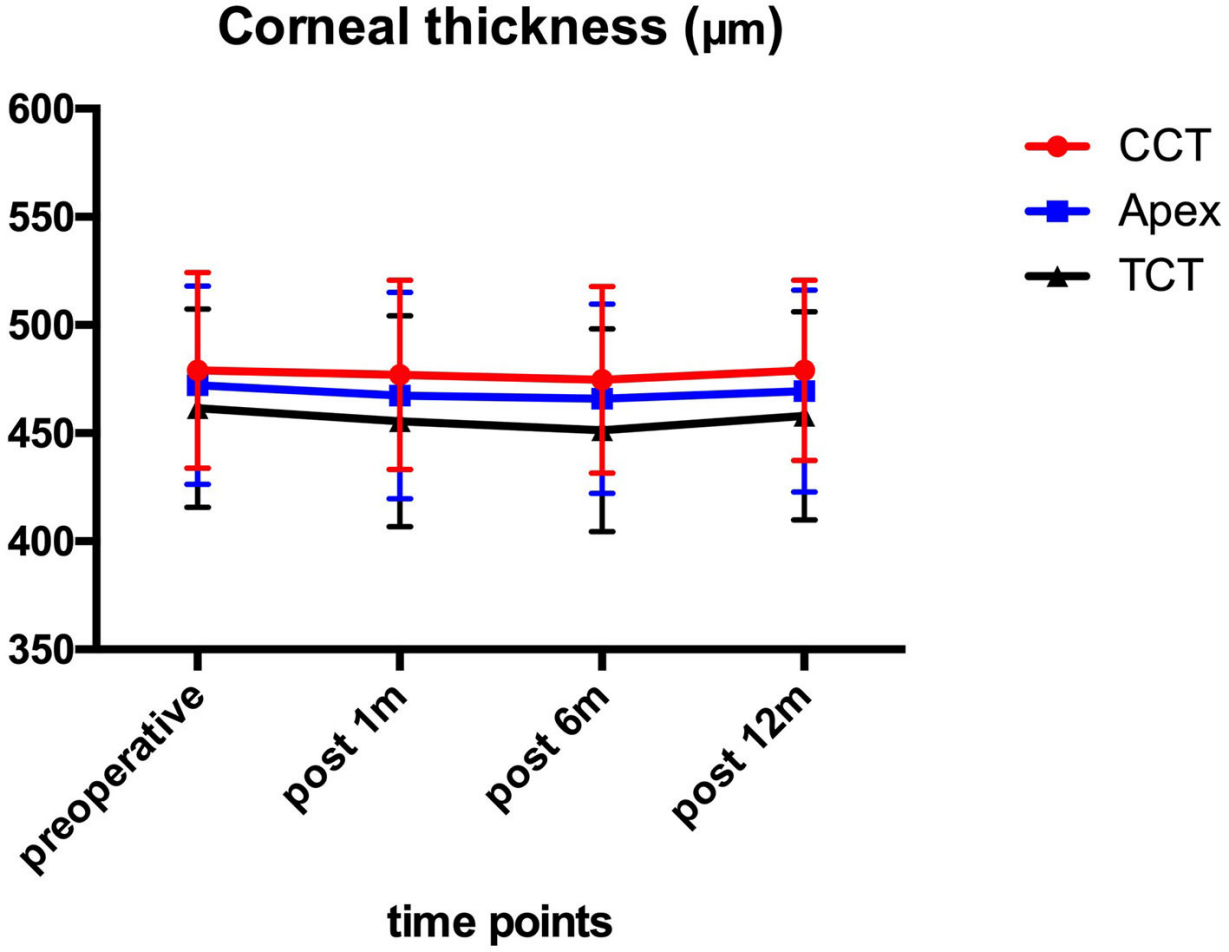
CCT, TCT, and AT before and after ATE-CXL during 1-year follow-up. No significant (*P* > 0.05) changes were found in CCT, TCT, and AT in comparison to preoperative values at each time point. (CCT, central corneal thickness; TCT, thinnest corneal thickness; AT, apex thickness; ATE-CXL, accelerated transepithelial corneal cross-linking).

Figure 2 shows the preoperative and post-operative corneal thickness. The CCT values were 479.10 ± 45.33 μm preoperatively, and 476.90 ± 43.83, 474.63 ± 43.28, and 479.10 ± 41.61 μm at 1, 6, and 12 month after the operation, respectively. No statistically significant (*P* > 0.05) changes were found for CCT, TCT, and AT values during the 1-year follow-up.

### DCR Parameters

The results of the corneal biomechanical properties assessment using the Corvis ST are illustrated in Table 3. The first applanation time (AT1), first applanation length (AL1), second applanation velocity (AV2), highest concavity time (HCT), and radius of curvature (Rad) increased significantly (*P* < 0.001, *P* = 0.004, *P* = 0.014, *P* = 0.022, and *P* = 0.031, respectively) in 1 year after ATE-CXL. Furthermore, The SP-A1 value significantly increased from 57.70 ± 27.57 preoperatively to 63.36 ± 27.09 at 1 year post-operatively (*P* = 0.018). There were no significant differences in other parameters between the preoperative and post-operative time points (*P* > 0.05).

**Table 3:** Pre-operative and post-operative parameters of Corvis ST (Mean ± SD).

	**preoperative**	**12 month**	***P* value**
AT1	6.56 ± 0.40	6.88 ± 0.34	<0.001^*^
AL1	1.85 ± 0.24	2.04 ± 0.37	0.004^*^
AV1	0.17 ± 0.03	0.18 ± 0.03	0.809
AT2	21.82 ± 0.59	21.90 ± 0.68	0.525
AL2	1.53 ± 0.42	1.56 ± 0.37	0.812
AV2	−0.33 ± 0.08	−0.31 ± 0.13	0.014^*^
HCT	16.47 ± 0.51	16.84 ± 0.70	0.022^*^
PD	4.97 ± 0.47	5.01 ± 0.26	0.465
Rad	4.64 ± 0.95	4.97 ± 0.86	0.031^*^
DA	1.27 ± 0.22	1.23 ± 0.19	0.097
SP-A1	57.70 ± 27.57	63.36 ± 27.09	0.018^*^
ARTH	210.12 ± 90.94	198.05 ± 90.23	0.168
DAR	6.44 ± 1.53	6.58 ± 1.87	0.381
Integr Rad	13.79 ± 3.12	13.27 ± 2.92	0.062

*AT1, first applanation time; AL1, first applanation length; AV1, first applanation velocity; AT2, second applanation time; AL2, second applanation length; AV2, second applanation velocity; HCT, highest concavity time; PD, peak distance; Rad, radius of curvature; DA, deformation amplitude; SP-A1, stiffness parameter at first applanation; ARTH, Ambrósio's relational thickness horizontal; DAR, deformation amplitude ratio; Integr Rad, integrated radius*.

* *Statistically significant*.

## Discussion

CXL is an effective treatment widely used to treat keratoconus. It is important to evaluate the efficacy of CXL by examination after the procedure, especially for pediatric patients with keratoconus because of the fast visual-developing period and the fact that any pathogenic effects may lead the irreversible vision loss. Corvis ST can be used to provide a series of DCR parameters in the diagnosis and treatment assessment of patients with keratoconus. In this study, we are the first to investigate the changes of corneal biomechanical properties in pediatric patients with progressive keratoconus after ATE-CXL.

Our results showed that the eyes remained stable with respect to Kmax 1 year after ATE-CXL when compared to the preoperative assessment, which may indicate that ATE-CXL is an effective treatment for halting the progression of pediatric keratoconus. Or et al. (7) and Eissa et al. (20) reported that Kmax remained stable 1 year after C-CXL and accelerated CXL (A-CXL) for pediatric patients with keratoconus, which was consistent with the results of our study.

The obtained results suggested no significant changes in CCT, TCT, and AT 1 year after ATE-CXL, which indicated the efficacy of stabilizing the corneal thickness in pediatric progressive keratoconus. Previous studies (6, 7, 21) assessing the C-CXL outcomes in pediatric patients with keratoconus reported a significant decrease in corneal thickness compared with preoperative results. In our study, the corneal thickness remained stable at each follow-up examination because the corneal epithelium was preserved during the operation.

In this study, no intraoperative or post-operative complications were observed during the follow-up, which indicated that ATE-CXL can be safely used to treat pediatric patients. It has been reported that (22–24) complications, such as corneal haze or infection were found in a short follow-up period after C-CXL treatment for keratoconus, but these complications did not occur in our study. The UVA radiation energy of the C-CXL treatment is lower, so the irradiation time is longer, and the patients need a longer recovery time due to the debridement of corneal epithelium. However, in this study, the process of ATE-CXL with epithelium-on procedure used higher UVA radiation energy, thus reducing the irradiation time, which not only allows patients to feel more comfortable during the treatment, but also let patients have shorter recovery periods after the treatment. Therefore, ATE-CXL is more suitable for pediatric patients with poor co-operation. Previous studies have postulated that A-CXL protocols with higher intensity UVA would result in more rapid oxygen depletion thereby reducing efficacy (25, 26). All eyes treated in our study received an application of pulsed light ATE-CXL, where oxygen availability theoretically should be greater than in the non-pulsed CXL treatments (27, 28). Mazzotta et al. (29) confirmed that pulsed light CXL treatment could produce better functional outcomes and a deeper stromal penetration than continuous light for keratoconus.

Our comparative analysis of the corneal biomechanical parameters obtained before and after ATE-CXL showed that AT1 and AL1 increased significantly during 1-year follow-up. Vinciguerra et al. (17) reported that AT1 increased from 6.74 ± 0.22 to 6.98 ± 0.54 in 34 eyes at 6 month after C-CXL, and Sedaghat et al. (30) reported that AL1 increased from 1.72 ± 0.33 to 2.08 ± 0.33 in 18 eyes after C-CXL in a 4-year follow-up study. However, these studies enrolled adult patients with keratoconus. AT1 represents the time when the cornea reached the first applanation. A stiffer cornea requires more time to reach the first applanation, so that the increase in AT1 indicates a significant increase in corneal stiffness. In this study, there was no change in DA at 1 year after ATE-CXL. DA represents the total displacement of the cornea from the original position at the highest corneal concavity time, so that the lower value represented the stronger resistance of cornea. Tomita et al. (31) found that DA significantly decreased in adult keratoconus after C-CXL. However, in a study of C-CXL by Sedaghat et al. (30) DA increased significantly in adult keratoconus at 4 years post-operatively, which indicated that our results needed to be confirmed in a longer follow-up. In addition, this study found that HCT increased significantly by 1 year after the operation, also suggesting the increase of corneal stiffness. HCT represents the time when the cornea reached the highest concavity, and healthier and stiffer corneas can more easily resist outside forces. Therefore, compared with eyes with keratoconus, a normal cornea needs more time to reach the highest concavity (32).

The deformation amplitude of a stiffer cornea is lower after being impacted by the pulse air, so the SP-A1 value is higher as detected by Corvis ST. Therefore, a healthy cornea shows a higher SP-A1 value compared with those with keratoconus (18). Another study also reported lower SP-A1 values in patients with more severe keratoconus (33). This study found that SP-A1 value increased significantly at 1-year post-operatively, which suggested that ATE-CXL can be used to increase corneal stiffness for pediatric keratoconus. In the observation of 66 adult cases of keratoconus Vinciguerra et al. (34) also found that SP-A1 was significantly increased at 1 month after CXL. In addition, the integrated inverse radius was also considered as a parameter for the evaluation of corneal stiffness, and it was lower in healthy corneas compared to corneas with keratoconus (35). In this study, there was no change in Integr Rad value at 1 year after ATE-CXL. However, Hashemi et al. (36) found that the Integr Rad significantly decreased in a 2-year follow-up study of 37 eyes after A-CXL. The possible reason is that the severity of keratoconus enrolled in our study is relatively higher, with the preoperative Kmax value is larger than that of the above-mentioned study (<55D). To our knowledge, this is the first study to report changes in corneal biomechanical properties of pediatric patients with keratoconus who were treated with ATE-CXL, which indicated that ATE-CXL could enhance the biomechanical strength of the cornea in pediatric keratoconus.

There were some limitations in this study. One was a relatively low number of patients, and another was the lack of a control group undergoing C-CXL. In addition, 1-year results of ATE-CXL is a short-term follow-up for pediatric patients. Further studies with a longer follow-up period to confirm the long-term biomechanical changes of the ATE-CXL treatment are also needed.

Our study suggests that ATE-CXL is a safe and effective treatment for pediatric patients with progressive keratoconus. The DCR parameters provided by Corvis ST can be used to evaluate changes of corneal biomechanics after cross-linking, which indicated that the corneal stiffness of pediatric keratoconus increased at 1 year after ATE-CXL, but the long-term effects need further observation.

## Data Availability Statement

The raw data supporting the conclusions of this article will be made available by the authors, without undue reservation.

## Ethics Statement

The studies involving human participants were reviewed and approved by Ethics Committee of the Eye and ENT Hospital of Fudan University. Written informed consent to participate in this study was provided by the participants' legal guardian/next of kin.

## Author Contributions

WJ, MT, and XZha were responsible for the initial plan, study design, data collection, data extraction, data interpretation, manuscript drafting, statistical analysis, and conducting the study. LS, YS, and ML were responsible for data collection, extraction, and critical revisions of the manuscript. WJ, MT, and XZho were responsible for data interpretation, manuscript drafting, supervision, and critical revisions of the manuscript for important intellectual content. XZho was the guarantor for this article and has full responsibility for this study. All authors contributed to the article and approved the submitted version.

## Conflict of Interest

The authors declare that the research was conducted in the absence of any commercial or financial relationships that could be construed as a potential conflict of interest. The handling editor is a consultant for the manufacturer Corvis ST, and declares that the assessment of the manuscript was conducted in the absence of any commercial or financial relationships that could be construed as a potential conflict of interest.
